# Diversity of social media use: Self-selection explains associations between using many platforms and well-being

**DOI:** 10.1371/journal.pdig.0000292

**Published:** 2023-07-13

**Authors:** Sophie Lohmann, Emilio Zagheni

**Affiliations:** Department of Digital and Computational Demography, Max Planck Institute for Demographic Research, Rostock, Germany; Tsinghua University, CHINA

## Abstract

Many people engage with a diverse array of social media platforms, raising concerns that this diversity of platforms may be linked to negative affect, hypothesized to arise from multitasking or identify diffusion. Using a large representative sample (*N* = 1,372) of US adults from the authoritative General Social Survey, we examine associations between social media diversity and well-being and propose a self-selection explanation for these associations. Even without accounting for selection bias, we find few and only small associations. Importantly, after using a rigorous propensity-score weighting technique to adjust for selection bias, these associations disappear. Further, we also document few negative associations between the use of specific social media platforms and well-being. Our findings suggest that (i) diverse social media use is not a major risk factor to adult well-being; (ii) negative correlations reported in the literature may be spurious; (iii) technology use research needs to take self-selection biases seriously.

## Author summary

Social media platforms like Facebook, Twitter, or Pinterest are not interchangeable. This is why it has been suggested that using many diverse platforms can have other effects than just spending a lot of time on a single platform. For example, people who use more diverse platforms may feel more overwhelmed because they have to multitask. On the other hand, they may also build more diverse relationships. We used a large, representative sample of US adults to look at whether diverse social media use is related to well-being. Overall, people who used more platforms were not happier or unhappier. The few effects that we found seemed to be due to self-selection. For example, people with worse mental health or who overall spend more time online may choose to use more platforms. When we took self-selection into account, these effects went away. Also, different platforms had very different associations with well-being. This again shows that different social media platforms cannot be treated the same.

## Introduction

Over the past 15 years, our lives have become intertwined with a multitude of social media platforms. Within just a few years, Facebook, Twitter, Instagram, LinkedIn, Pinterest, and recently TikTok have gone from virtually unknown to household names. For example, 69% of the US population now uses Facebook, 28% use Pinterest, and Instagram use recently reached 37% [1]. Similar trends have occurred globally, and an estimated 3.8 billion people worldwide, almost half the global population, use some form of social media [2]. Consequently, commentators and researchers have been keenly interested in whether the use, the over-use, or the time spent on any of these diverse platforms affects our mental and physical health. However, due to a lack of research, there is no conclusive evidence on whether this diversity itself is helpful or harmful.

We define social media diversity as the number of social media platforms a person uses. Even though they are often all summarized as “social media” and even sometimes treated as substitutable, different platforms have very different technical features, user bases, and community cultures [3]. The experience of being exposed to only one platform (and therefore one set of features, one user base, and one community culture) can therefore differ vastly from the experience of someone who participates in many different platforms. Additionally, the latter person likely uses social media in more areas of their life (e.g., Facebook to keep in touch with friends and family, Twitter to read and share news, Pinterest to find new recipes, and LinkedIn to connect with business contacts). This mechanism is important to study because individual platforms can come and go (see the deactivation of Google+ and the emergence of TikTok), but the underlying question of how the access to a diverse array of social media platforms shapes our lives remains.

Based on theoretical principles and the results of two previous studies [4,5], we assume that social media diversity should be correlated with, but distinct from time use: On average, people who use many platforms likely spend more time on social media, but somebody who uses just one platform extensively may spend just as much time. Several highly-publicized studies have found detrimental effects of time spent on social media on depression and other indicators of well-being, especially among adolescents and young adults [6–8]. Later re-analyses of these datasets as well as analyses of new large datasets, however, revealed only very small effects or even null effects [9–13]. Meta-analyses of this literature have confirmed the latter findings by showing that, on average, associations between time spent on social media and well-being are no larger than *r* = -.10 [14–16].

Since the field has started to tentatively converge on the conclusion that overall time spent on social media is only minimally related to well-being among adults, research has instead shifted to identifying particular characteristics of people or particular characteristics of social media usage that may pose a stronger risk to well-being [e.g., 17]. An important recent study hints at this possibility by showing substantial interindividual variability: 46% of adolescents actually felt more positive after using social media, whereas 10% felt worse and the rest did not feel any different than before [18]. It is therefore likely that several ways of using social media are more harmful than others. For example, the distinction between active and passive use has received much attention and the literature finds that passively consuming social media is typically associated with lower well-being, whereas actively creating and posting content is not [19,20, however, see also 21, and 22]. One particular characteristic of social media usage that has been proposed as a risk factor [4,5,23], but has not received much research attention yet, is social media diversity.

How social media diversity relates to well-being is an unanswered question and there exists no consensus on which characteristics of social media diversity may threaten or promote well-being. There are several theoretical pathways which would predict either negative or positive effects. The few existing empirical explorations of social media diversity have linked it to negative affect, hypothesized to arise from multitasking [4,5,23], from increased opportunities for social comparison which are known to be detrimental to well-being [24], and to identity diffusion [4]. First, however, it may also hold positive potential: It may help people better tailor their online experiences to their needs by allowing access to more features [e.g., communicating with the public under a pseudonym on Twitter, while also leading self-deleting one-on-one conversations with friends on Snapchat, 25,26]. Each new platform also offers users a new context in which to meet people, and having social ties in a variety of contexts (social integration), has been found to have health-protective effects along several dimensions, from susceptibility to the common cold to suicidality [27–29]. Most of these studies have focused on offline social contexts, but in the online realm, it has also been established that increased social capital from social media platforms can have beneficial effects on well-being [30].

Second, much of the influence of social media diversity on well-being might be accounted for by self-selection effects. People self-select which platforms they want to use, which can bias the data if the reasons for their choices are also related to their well-being. If people choose to use many platforms based on some socio-demographic characteristics [e.g., younger age or lower socio-economic status, 1], but these characteristics also predispose them to lower well-being [e.g., young adults experience higher rates of mental illness than older adults, 31,32; and low socio-economic status predicts lower well-being, 33], we may see an association between multi-platform use and well-being that is negative, but spurious. Similarly, people who generally spend much time online—whether due to addiction-like usage [34], attempts to compensate for offline loneliness [35], or another reason—will likely self-select into using a diverse array of social media. These are examples of self-selection effects based on demographic characteristics or based on general internet use that can therefore bias the data. Similar results have been found regarding social media time use, where people with pre-existing mental health problems are more likely to use social media more intensively, explaining at least part of the negative association between social media time use and well-being [21,36–38]. Without accounting for selection bias, we cannot draw firm conclusions about the effects of social media use. Nonetheless, past work in this literature has not properly considered selection effects. A very small number of studies has attempted to experimentally study the effects of social media use on well-being [39–44]. However, questions of cumulative effects across years and across multiple platforms are next to impossible to study experimentally: For example, it would neither be ethical nor feasible to require participants to spend two hours each day on Twitter and Instagram for the next three years while not visiting any other social media sites. For this reason, the vast majority of studies in this area has used correlational survey data and, while sometimes mentioning self-selection biases as a limitation, did not address it methodologically [e.g., 45,46].

Our contribution with this paper is twofold: First, we introduce a theoretical account of the so-far under-researched concept of social media diversity and its association with well-being. Second, we propose a self-selection explanation of this association based on demographic characteristics as well as general internet use and provide the first test of such self-selection effects in the social media use literature. We use a nationally representative sample to first examine the prevalence of diverse social media use and define the population of high-diversity users. Next, we study the effects of diverse social media use across the full range of well-being indicators (including mental health, physical health, and social well-being) and control for self-selection effects through rigorous use of propensity-weighting techniques. Finally, because the average effect of using many platforms may obscure contradictions when two different platforms have opposing effects that cancel each other out, we also present platform-specific effects on well-being. This paper therefore answers three research questions:

RQ 1: Who is likely to use diverse social media platforms?
RQ 2: How is diverse social media use related to well-being (a) overall and (b) after accounting for self-selection?
RQ 3: How are specific social media platforms related to well-being?

To answer these questions, we drew on nationally representative data from the General Social Survey [GSS; 47], *N* = 1,372 US American adults.

## Results

### Influence of Socio-Demographic factors on social media diversity

First, we established how the socio-demographic factors that we considered as possible self-selection variables predicted how many platforms people were using (RQ 1). A linear regression (Table 1) showed that respondents tended to use a more diverse set of platforms when they were younger, when they had been using the internet for longer, and when they spent more time using the internet on weekends (but not weekdays). In addition, women, people from higher socio-economic backgrounds, and those with a recent history of migration in their family used more diverse platforms. Finally, the living environment appeared to play a role as well: People living in smaller households or larger cities and those living outside of the largely rural and sparsely populated West North Central region of the US (see map in Figure C in S1 Appendix) tended to use the most platforms. Next, we used these results to form a propensity score with which we adjusted the influence of social media diversity on well-being to rule out self-selection effects from common third variables, such as age or socio-economic status.

**Table 1:** Linear regressions of socio-demographic predictors on number of social media platforms.

	Model 1			Model 2		
Term	*b*	*p*	95% CI	*b*	*p*	95% CI
(Intercept)	4.38*	< .001	[3.97, 4.80]	3.90*	< .001	[3.47, 4.32]
City size	0.00	.053	[-0.00, 0.00]	0.00*	.010	[0.00, 0.00]
Household size	-0.09*	.046	[-0.18, -0.00]	-0.04	.393	[-0.13, 0.05]
Gender: Male	-0.74*	< .001	[-0.96, -0.51]	-0.62*	< .001	[-0.84, -0.40]
Age	-0.05*	< .001	[-0.06, -0.04]	-0.03*	< .001	[-0.04, -0.02]
Nr of children	-0.04	.397	[-0.13, 0.05]	-0.05	.324	[-0.14, 0.04]
Race/Ethn.: White (ref.)	0.00			0.00		
Hispanic	-0.30	.162	[-0.71, 0.12]	-0.33	.114	[-0.75, 0.08]
Non-Hispanic Black	0.19	.302	[-0.17, 0.56]	0.10	.598	[-0.27, 0.46]
Non-Hispanic Other	0.55	.058	[-0.02, 1.12]	0.28	.318	[-0.27, 0.83]
SES index	0.30*	.003	[0.10, 0.49]	0.35*	< .001	[0.16, 0.55]
Foreign family index	0.18*	.021	[0.03, 0.33]	0.23*	.003	[0.08, 0.38]
Religiosity index	0.12	.089	[-0.02, 0.26]	0.06	.366	[-0.08, 0.20]
Political conservatism index	-0.07	.324	[-0.20, 0.07]	-0.06	.386	[-0.19, 0.08]
Internet use start (centered at 2000)	-0.04*	< .001	[-0.06, -0.02]	-0.03*	< .001	[-0.05, -0.02]
Region: East North Central (ref.)	0.00			0.00		
East South Central	-0.18	.496	[-0.70, 0.34]	0.00	.992	[-0.52, 0.52]
Middle Atlantic	0.02	.910	[-0.40, 0.44]	-0.01	.979	[-0.41, 0.40]
Mountain	-0.42	.071	[-0.88, 0.04]	-0.41	.073	[-0.86, 0.04]
New England	-0.32	.229	[-0.83, 0.20]	-0.02	.954	[-0.54, 0.51]
Pacific	-0.26	.188	[-0.66, 0.13]	-0.10	.613	[-0.49, 0.29]
South Atlantic	-0.09	.623	[-0.46, 0.27]	-0.03	.866	[-0.39, 0.33]
West North Central	-0.70*	.008	[-1.21, -0.19]	-0.52*	.047	[-1.04, -0.01]
West South Central	-0.06	.812	[-0.52, 0.41]	0.15	.514	[-0.31, 0.61]
Internet use time on weekdays				0.03	.217	[-0.02, 0.07]
Internet use time on weekends				0.08*	< .001	[0.03, 0.12]

* *p* < .05

### Influence of social media diversity on well-being

We first present a series of simple, unadjusted linear OLS regressions of social media diversity on well-being (left section of Table 2; RQ 2a). Even without accounting for selection effects, we find few effects and small effect sizes on adult well-being. On the positive side, people who used more platforms reported finding life more exciting, *b* = 0.04 (i.e., one additional platform means an increase of 0.04 *SD*s) and having more confidence in societal institutions, *b* = 0.03; on the negative side, they were more likely to have ever felt like they were going to have a nervous breakdown, *b* = 0.06. It was this latter item that led previous analysts to conclude that social media negatively affects well-being [23]. However, this item had questionable validity because a breakdown was ill-defined and the item did not show strong concurrent validity with other well-being indicators. The question did not explain to respondents what a “nervous breakdown” meant, likely leading to considerable variation in subjective definitions. This item showed only low-to-medium correlations with all other well-being indicators in the survey (e.g., happiness: *r* = -.14, health: *r* = -.17, depression: *r* = .31, bad mental health days: *r* = .25). The item was, however, still included in the analyses to show a complete index of available well-being measures without selective reporting and for comparison with a previous study, which had used this item as their only outcome measure to argue that social media diversity is harmful [23]. Further, it was the only item referring to respondents’ entire lifespan instead of current well-being, making it less than optimal for the analysis of recent phenomena (such as the widespread adoption of social media) and current well-being. If interpreting this result as non-spurious, it could therefore suggest potential reverse causation: If lifespan mental health relates to social media use but current mental health does not, the most likely explanation is that earlier well-being deficits led people to self-select into social media instead of social media affecting well-being.

**Table 2:** Results of unadjusted and propensity-adjusted IPTW regressions of social media diversity on z-standardized well-being indicators. IPTW-adjusted estimates represent means and 95% percentiles of 5,000 bootstrapped samples. Maximum N = 1372 for unadjusted models, 1161 adjusted for demographic selection effects, and 1042 adjusted for demographic and internet use effects.

		Unadjusted		IPTW-adjusted for demographics		IPTW-adjusted for demographics plus internet time use	
Outcome (standardized)	Original range	*b*	95% CI	*b*	95% CI	*b*	95% CI
Happiness	-1 to 1	0.00	[-0.02, 0.03]	-0.02	[-0.08, 0.02]	-0.02	[-0.09, 0.03]
Health	-1 to 2	0.01	[-0.03, 0.05]	-0.03	[-0.10, 0.04]	0.03	[-0.08, 0.08]
Depression	0 to 3	0.02	[-0.02, 0.05]	0.07*	[0.00, 0.15]	0.03	[-0.03, 0.16]
Bad mental health days	0 to 30	0.03	[-0.00, 0.06]	0.08	[-0.01, 0.28]	0.01	[-0.04, 0.07]
Ever breakdown	0 to 1	0.06*	[0.03, 0.10]	0.06*	[0.00, 0.11]	0.03	[-0.04, 0.08]
Excitement about life	-1 to 1	0.04*	[0.00, 0.08]	0.01	[-0.17, 0.08]	0.01	[-0.20, 0.10]
Financial satisfaction	-1 to 1	-0.01	[-0.03, 0.02]	-0.03	[-0.08, 0.01]	0.00	[-0.04, 0.04]
Relationship satisfaction	-1 to 1	0.03	[-0.01, 0.07]	0.03	[-0.02, 0.07]	0.03	[-0.03, 0.08]
Social trust	-1 to 1	0.00	[-0.03, 0.02]	0.02	[-0.05, 0.08]	0.04*	[0.00, 0.10]
Confidence in soc. institutions	-1 to 1	0.03*	[0.00, 0.05]	0.05*	[0.00, 0.13]	0.03	[-0.03, 0.13]

* Confidence interval did not include 0

Next, we used inverse-probability of treatment weighting (IPTW) for continuous independent variables, a propensity score adjustment method [48–50], to understand selection biases, and partially correct for them in our estimates. IPTW is used when assignment to an independent variable was non-random based on selection characteristics (e.g., age and income), but analysts wish to estimate the independent variable’s effect if assignment had been random (at least with respect to these observed characteristics). IPTW has been successfully and widely used in the medical sciences [51,52], but has been largely ignored in the social media literature despite its potential for improving causal effect estimates in correlational data which are skewed by self-selection bias. IPTW offers an approach for controlling for a portion of this bias and can thus help disentangle spurious and substantive effects.

**Fig 1:**
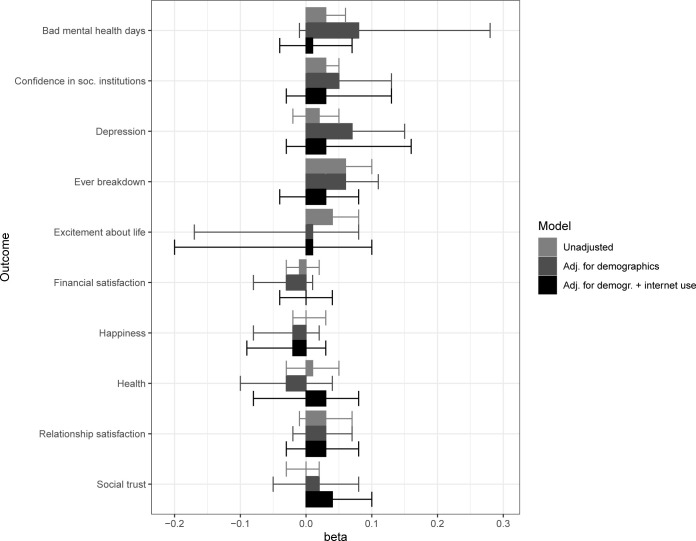
Comparison of average bootstrapped beta coefficients from the regressions of social media diversity on well-being outcomes (visualization of the same values as in Table 2).

We now present findings after applying this IPTW procedure (RQ 2b; Fig 1). After adjusting for the propensity to use multiple platforms based on demographic variables, the positive effect of social media diversity on finding life more exciting decreased to almost zero, *b* = 0.01, *ns*. The remaining two effects appeared to be accounted for by time use self-selection factors, such that people who generally spend more time on the internet also use more platforms: After additionally including weekday and weekend time use in the calculation of the propensity score, the negative effect of social media diversity on ever having felt like having a breakdown was halved and became non-significant, *b* = 0.03. The positive association with increased social confidence also became non-significant due to increased variance introduced by the IPTW procedure and bootstrap, although the effect size remained the same, *b* = 0.03. After accounting for self-selection involving both demographic and time use variables, the only remaining effect was a positive influence of social media diversity on increased social trust, *b* = 0.04, which had not been visible in the unadjusted results.

### Influence of specific social media platforms on well-being

Finally, we analyzed if any specific social media platforms were associated with personal or social well-being when controlling for the same socio-demographic characteristics as in the previous analyses as well as all other social media platforms (Table 3; RQ 3). Again, all well-being indicators were z-standardized to facilitate comparison of effect sizes between indicators. Even after adjusting for these various controls, Tumblr users still reported pronouncedly lower well-being: Lower happiness (*b* = .49 [-.75, -.22], *p* = .001), lower health (*b* = -.55 [-.92, -.17], *p* = .009), and lower confidence in social institutions (*b* = -.35 [-.58, -.12], *p* = .006). Tumblr is known as a platform with strong activist communities around mental health and physical disability where such problems are prominently discussed or turned into communal coping humor. It is therefore possible that users with physical or mental health problems are particularly drawn to this platform. Further, Pinterest users were more likely to report ever having felt like having a breakdown (*b* = .25 [.06, .45], *p* = .018). All other associations, however, were either non-significant or positive, such as LinkedIn users reporting better physical health (*b* = .25 [.10, .40], *p* = .003) and Snapchat users reporting fewer bad mental health days (*b* = -.26 [-.43, -.08], *p* = .009).

**Table 3:** Results of weighted linear regressions predicting z-standardized measures of well-being from social media platforms and socio-demographic controls.

	Happiness		Health		Depression		Bad mental health days		Ever breakdown		Excitement		Financial satisf.		Relationship satisf.		Trust		Confidence	
Term	*b*	*p*	*b*	*p*	*b*	*p*	*b*	*p*	*b*	*p*	*b*	*p*	*b*	*p*	*b*	*p*	*b*	*p*	*b*	*p*
Facebook	0.00	.979	-0.22	.061	0.05	.594	0.02	.823	0.08	.458	-0.13	.172	-0.09	.265	0.06	.551	0.10	.188	0.14*	.050
Twitter	-0.01	.904	-0.02	.865	-0.07	.439	-0.01	.952	-0.25	.056	0.11	.266	0.14	.132	0.00	.971	0.05	.604	0.15*	.049
Tumblr	-0.49*	.001	-0.55*	.008	0.26	.407	0.10	.575	0.24	.302	-0.32	.192	-0.22	.179	-0.31	.195	-0.08	.512	-0.35*	.006
Snapchat	0.15*	.041	0.18	.148	-0.12	.249	-0.26*	.009	-0.01	.928	0.20	.088	0.13	.192	0.15	.167	-0.01	.913	0.09	.339
Vine	0.09	.434	-0.24	.326	0.03	.858	0.02	.903	-0.05	.791	-0.32	.156	0.13	.429	0.23	.098	-0.09	.384	0.27	.069
Instagram	-0.13	.127	-0.11	.280	0.17	.104	0.09	.405	-0.05	.684	-0.03	.761	0.00	.993	0.08	.464	-0.04	.546	-0.11	.182
Pinterest	0.13	.126	-0.02	.849	-0.10	.264	0.02	.849	0.25*	.018	0.15	.088	-0.01	.898	0.02	.839	0.06	.365	0.01	.935
Flickr	0.18	.425	0.19	.395	-0.03	.867	0.20	.449	0.30	.281	0.23	.315	-0.19	.370	0.30	.307	-0.08	.702	0.23	.343
WhatsApp	0.00	.970	0.25	.065	0.16	.208	0.04	.767	0.17	.354	0.13	.275	0.00	.971	0.03	.839	-0.02	.849	-0.11	.297
Classmates	-0.20	.196	-0.27	.222	0.19	.327	0.20	.312	0.14	.481	0.02	.926	-0.18	.332	-0.25	.152	-0.08	.591	0.01	.936
LinkedIn	-0.04	.661	0.25*	.003	0.03	.721	0.04	.665	0.07	.503	-0.07	.442	-0.1	.121	-0.07	.490	0.07	.368	-0.11	.180
Google+	0.00	.989	0.06	.556	-0.03	.753	0.00	.978	0.06	.493	-0.04	.606	-0.05	.477	0.09	.368	0.08	.260	0.05	.468
Demographic controls (not printed here) ^a^	. . .	. . .	. . .	. . .	. . .	. . .	. . .	. . .	. . .	. . .	. . .	. . .	. . .	. . .	. . .	. . .	. . .	. . .	. . .	. . .

* *p* < .05

^a^ The coefficients for the demographic control variables are omitted for brevity, see Table D in S1 Appendix for the complete table.

## Discussion

To answer our first research question of who is likely to use diverse social media platforms (RQ 1), we found that diverse social media use was more frequent in younger people, women, people of higher socio-economic status, from foreign-born families, and in larger cities. Further, those who started using the internet earlier and who spent more time using the internet on weekends tended to use more platforms. In examining effects of social media diversity on adult well-being and examining a self-selection hypothesis (RQ 2) in a large, nationally representative sample, we find that associations between social media diversity or individual social media platform use and adult well-being are (1) rare and inconsistent across operationalizations of well-being, (2) small when they occur, and (3) heavily driven by self-selection effects. Both negative (having ever felt like having a breakdown) and positive (more excitement about life, more confidence in society) associations disappeared when adjusting for the propensity to be using many social media platforms in the first place, based on demographic factors and general internet use. The only association that emerged after these adjustments was positive in valence, suggesting that people who use more diverse social media platforms also report higher trust in others. In short, we observed a third-variable effect: In unadjusted analyses, social media diversity and well-being appeared negatively related, but this was an artefact of underlying demographic and internet use variables, which produced a spurious association. Finally, there were no consistent effects when examining individual social media platforms (RQ 3). Most platforms were unrelated to most forms of well-being, with the notable exception of Tumblr use, which was consistently related to poorer mental and physical health. Our results shine a new light on the small number of previous analyses of social media diversity and well-being [4,5,23,24] by suggesting that the negative effects that they found may be in large part due to self-selection effects.

It is worth noting that none of the effects, positive or negative, unadjusted or adjusted, significant or not, were sizeable. For example, the likelihood of ever having felt like having a breakdown was increased by only 6% of a standard deviation when using one additional social media platform (strongest effect in our unadjusted analyses) and social trust increased by only 4% of a standard deviation (strongest effect in our fully adjusted analyses). These generally low effect sizes match results of social media time use on well-being [e.g., meta-analytic r = -0.07, 53,14,9,15,10,54]. It may therefore be hard to observe noticeable, practically relevant differences in an individuals’ well-being unless they are using very many platforms, for example, eight platforms instead of the sample median of three.

Because the social media module was included in the GSS only once to date, this dataset offers only a cross-sectional snapshot. It is possible that, at the macro-level, population well-being changed when a diverse array of social media platforms first reached widespread popularity or that, at the micro-level, increases or decreases in social media diversity change well-being. However, such a historical analysis is beyond the scope of the current paper. Because the only wave of the GSS that included the social media module is from 2016, the data are additionally several years old at this point and patterns may have changed. We encourage the GSS to add this module to a future wave, given that the importance of social media to society has not lessened since 2016. That said, our study may be more robust to changes in the social media landscape than other studies precisely because our key results do not rely on the characteristics of any single platform, but rather on the variety of platforms that exist. Individual platforms coming and going might therefore not be expected to lead to major changes in the patterns we observed. On the other hand, new developments like the convergent evolution of several platforms (such as the increasing use of shorts or reels on many platforms including TikTok, Youtube, Instagram, Reddit, and even Spotify) could be speculated to decrease the variety in the social media landscape overall. We encourage future research to take up this question.

Even though our findings in this article cannot speak to such macro-level developments, they offer relevant and timely information going forward: With diverse social media already a ubiquitous part of everyday life and interpersonal communication patterns, should people avoid most social media platforms to overall improve population well-being? The present results suggest that this step may not be necessary because the number of used social media use is not consistently related to poorer (or better) well-being. Future studies should collect longitudinal data to extend these findings to intra-individual changes over time. Such longitudinal data would also be valuable to test the direction of associations through cross-lagged paths: One possible explanation of the selection effects we found is that while diverse social media use does not influence well-being, but that prior well-being could have influenced diversity of current social media use. Our current cross-sectional data do not allow us to test this possibility directly, but future longitudinal data could.

Although the current dataset was based on a representative sample, it included only US residents and the extent to which results transfer to other structural and cultural contexts is an empirical question that future work should examine. Finally, our results represent an average across the population, and future research may uncover more diverse underlying effects. For example, social media diversity may have negative consequences in some circumstances [such as when the choice to use many platforms was driven by peer pressure and fear of missing out, 55], but positive consequences in others [such as when it is perceived to be an autonomous choice to reap the multiple benefits that different platforms offer, 56]. Typically, there is a tradeoff in data sources in this field: Studies that aim for high explanatory depth by assessing how exactly people use each social media platform, which content they’re exposed to, and how they subjectively perceive this use usually focus on just a single or, at most, a handful of platforms and a smaller sample size to keep the study manageable. Conversely, studies that aim for wide coverage of different platforms or for a large, representative sample usually ask only surface-level questions about their use to keep the study manageable. In this study, we chose the GSS, which falls into the latter category, because our goal was to assess the full span of social media diversity and to obtain generalizable results. This choice means that we know how many platforms people are using, but future research is needed to fill in how they are using them.

In sum, we document few associations between the diversity of social media platforms that US adults use and their physical, mental, or social well-being. When associations with specific platforms persisted after controlling for socio-demographic factors, they were most often positive in nature, with Tumblr as the strongest exception. The strongest influences on how many social media platforms someone was using were whether they were young, female, and of high socio-economic status, as well as how long and how often they had been using the internet. We presented the first analysis of self-selection effects on social media diversity and find that how many platforms someone uses depends on who they are and how much they use the internet in general. These factors may, in turn, influence their happiness, but after taking these influences into account, how many different types of social media we are using does not appear to determine whether we are happy or unhappy.

## Materials and methods

We use the 2016 wave of the General Social Survey [GSS; 47] which asked *N* = 1,372 respondents about their social media use. The GSS comprises predominantly face-to-face interviews (English or Spanish) targeting adults living in households in the US. Participants were, on average, *M* = 42.94, *SD* = 16.00 years old, ranging from 18 to 89+ (coded as 89); 56% were women, and 69% identified as Non-Hispanic White (14% Non-Hispanic Black, 13% Hispanic, 5% Other). Because not all questions were shown to all respondents, sample sizes differ by dependent variable (see Table A in S1 Appendix). However, even the sparsest variable had enough participants (*n* = 680) to detect effect sizes of *r* ≥ .11 with 80% power. The GSS uses an area-probability cluster sample with two-stage sub-sampling for nonresponse with weights and is designed to produce results that are representative of the US population when taking survey weights into account. Our results are therefore based on design-corrected standard errors that incorporate design weights and nonresponse weights. The original data collection was approved by the NORC Institutional Review Board and details of the ethics compliance can be found in the GSS documentation at https://gss.norc.org/. Data was collected only from adults aged 18 or older. As a form of secondary data analysis, our study was IRB-exempt. The full analysis script and the Supplemental Materials can be found at https://osf.io/d9xn2/.

### Variables

#### Social media diversity

Respondents indicated whether they used Twitter, Facebook, Instagram, LinkedIn, Snapchat, Tumblr, WhatsApp, Google+, Pinterest, Flickr, Vine, and Classmates, plus optional free-form responses (we excluded gaming, dating sites, and Spotify from these responses). Values were treated as missing for people who reported not using the internet at all. By summing these binary variables, we derived a continuous index of social media diversity (0–14, *M* = 2.88, *SD* = 2.06).

#### Personal well-being

The survey included several indicators of subjective well-being: Happiness (1 = *very happy*, 0 = *pretty happy*, -1 = *not too happy*), physical health (2 = *excellent*, 1 = *good*, 0 = *fair*, -1 = *poor*), and excitement about life (*In general*, *do you find life* 1 = *exciting*, 0 = *pretty routine*, *or* -1 = *dull*?). Although these questions provide only a short subjective assessment, prior research has found that such subjective single-item questions are strongly predictive of future outcomes, including mortality [57,58]. Another question asked participants about their financial satisfaction (1 = *satisfied*, 0 = *more or less*, -1 = *not at all*).

Mental health was assessed with a 5-item version of the Center for Epidemiologic Studies Depression (CES-D) Scale (feeling depressed, happy (reverse-coded), lonely, sad, and experiencing restless sleep during the preceding week). Responses ranged from 0 = *none or almost none of the time* to 3 = *all or almost all of the time* and formed an internally consistent index, Cronbach’s α = .76, and were thus averaged into a composite score. Respondents also indicated how many days in the past month their mental health had not been good (0–30), as well as whether they had ever felt like they were going to have a nervous breakdown (0 = *no*, 1 = *yes*).

#### Social well-being

Relationship satisfaction was assessed by whether participants considered their marriage or partnership a happy one (1 = *very happy*, 0 = *pretty happy*, -1 = *not too happy*). Respondents also indicated whether they considered people in general helpful (1) vs. looking out for themselves (-1), fair (1) vs. likely to try and take advantage (-1), and trustworthy (1) vs. “can’t be too careful” (-1). These items were averaged to obtain a composite index of social trust, Cronbach’s α = 0.65. In addition, 13 items asked respondents how much confidence they had (1 = *a great deal*, 0 = *only some*, -1 = *hardly any*) in societal institutions, such as organized religion, education, television, or congress. These variables were combined into an index of social confidence, Cronbach’s α = 0.78.

### Socio-demographic characteristics

Respondents indicated their gender, age (the oldest participants were identified as “89 or older”, which we counted as 89), how long they had been using the internet, race (White, Black, Other) and ethnicity (Hispanic/Latino/Latina or not), household size, and number of children. The interviewer coded the approximate size and region of the respondent’s place of living. We further included an index of socio-economic status (standardized and averaged: income, occupational prestige, employed vs. not, full-time employed vs. not, college degree, subjective social rank recoded so 1 = *bottom* and 10 = *top*), an immigration index (number of foreign-born grandparents, whether at least one parent was foreign-born, whether participant was born abroad), a religiosity index (strength of religious affiliation, frequency of attending worship, frequency of attending other religious events), and an index of political conservatism (self-rated conservatism and whether the participant had or would have voted conservative in the 2012 election).

In addition to self-selection based on demographic factors, we consider self-selection based on general internet use. Participants reported how many hours they spent online on a typical weekday, *M* = 3.13, *SD* = 3.43, and a typical weekend day, *M* = 2.93, *SD* = 3.42. The two variables correlated substantially with each other, *r* = .63, but only weakly with social media diversity, *r* = .18 [.13, .24] and *r* = .20 [.14, .27], respectively.

### Analysis

IPTW [48–50] was used to control for selection effects. In IPTW, participants are re-weighted to generate a pseudo-population in which everyone, regardless of their selection characteristics, is equally likely to have a specific level of the independent variable. First, we calculated propensity scores by obtaining the fitted probabilities from a regression in which social media diversity was regressed on the socio-demographic variables (results in S1 Appendix). In a second step, we used these propensity scores as inverse-probability of treatment weights when regressing well-being measures on social media diversity. We combined these newly-calculated weights with the GSS survey weights by multiplying them [59]. To calculate standard errors and confidence intervals, this procedure was bootstrapped with 5,000 replicates; we computed bias-corrected and accelerated confidence intervals based on these results. The well-being outcomes were z-standardized for easier comparison.

## Supporting information

S1 Appendix All analyses from the manuscript as well as additional analyses, direct output from *rmarkdown/knitr* to verify results reported in text. (PDF)

S2 Appendix Codebook of all variables names, definitions, and recoding procedures used from/applied to the GSS dataset. (PDF)

## References

[1] Perrin A, Anderson M. Share of U.S. adults using social media, including Facebook, is mostly unchanged since 2018 [Social media update 2019]. Pew Research Center; 2019. Available: https://www.pewresearch.org/fact-tank/2019/04/10/share-of-u-s-adults-using-social-media-including-facebook-is-mostly-unchanged-since-2018/

[2] we are social, Hootsuite. Digital 2020: 3.8 billion people use social media. 2020. Available: https://wearesocial.com/blog/2020/01/digital-2020-3-8-billion-people-use-social-media

[3] boyd danah m, Ellison NB. Social network sites: Definition, history, and scholarship. Journal of Computer-Mediated Communication. 2007;13: 210–230. doi: 10.1111/j.1083-6101.2007.00393.x

[4] Primack BA, Shensa A, Escobar-Viera CG, Barrett EL, Sidani JE, Colditz JB, et al. Use of multiple social media platforms and symptoms of depression and anxiety: A nationally-representative study among U.S. young adults. Computers in Human Behavior. 2017;69: 1–9. doi: 10.1016/j.chb.2016.11.013 PMC13268370 42305949

[5] Vannucci A, Ohannessian CM, Gagnon S. Use of Multiple Social Media Platforms in Relation to Psychological Functioning in Emerging Adults. Emerging Adulthood. 2019;7: 501–506. doi: 10.1177/2167696818782309

[6] Kross E, Verduyn P, Demiralp E, Park J, Lee DS, Lin N, et al. Facebook Use Predicts Declines in Subjective Well-Being in Young Adults. PLOS ONE. 2013;8: e69841. doi: 10.1371/journal.pone.0069841 23967061 PMC3743827

[7] Twenge JM, Joiner TE, Rogers ML, Martin GN. Increases in Depressive Symptoms, Suicide-Related Outcomes, and Suicide Rates Among U.S. Adolescents After 2010 and Links to Increased New Media Screen Time. Clinical Psychological Science. 2018;6: 3–17. doi: 10.1177/2167702617723376

[8] Twenge JM, Campbell WK. Media Use Is Linked to Lower Psychological Well-Being: Evidence from Three Datasets. Psychiatr Q. 2019;90: 311–331. doi: 10.1007/s11126-019-09630-7 30859387

[9] Coyne SM, Rogers AA, Zurcher JD, Stockdale L, Booth M. Does time spent using social media impact mental health?: An eight year longitudinal study. Computers in Human Behavior. 2020;104: 106160. doi: 10.1016/j.chb.2019.106160

[10] Orben A, Dienlin T, Przybylski AK. Social media’s enduring effect on adolescent life satisfaction. Proceedings of the National Academy of Sciences. 2019;116: 10226–10228. doi: 10.1073/pnas.1902058116 31061122 PMC6534991

[11] Orben A, Przybylski AK. Screens, Teens, and Psychological Well-Being: Evidence From Three Time-Use-Diary Studies. Psychological Science. 2019;30: 682–696. doi: 10.1177/0956797619830329 30939250 PMC6512056

[12] Puukko K, Hietajärvi L, Maksniemi E, Alho K, Salmela-Aro K. Social Media Use and Depressive Symptoms—A Longitudinal Study from Early to Late Adolescence. IJERPH. 2020;17: 5921. doi: 10.3390/ijerph17165921 32824057 PMC7459880

[13] Sewall C, Goldstein TR, Wright AGC, Rosen D. Does objectively-measured social media or smartphone use predict depression, anxiety, or social isolation among young adults? PsyArXiv; 2021 Jul. doi: 10.31234/osf.io/ucsh6 PMC9671480 36406004

[14] Appel M, Marker C, Gnambs T. Are social media ruining our lives? A review of meta-analytic evidence. Review of General Psychology. 2020;24: 60–74. doi: 10.1177/1089268019880891

[15] Huang C. Time Spent on Social Network Sites and Psychological Well-Being: A Meta-Analysis. Cyberpsychology, Behavior, and Social Networking. 2017;20: 346–354. doi: 10.1089/cyber.2016.0758 28622031

[16] Yoon S, Kleinman M, Mertz J, Brannick M. Is social network site usage related to depression? A meta-analysis of Facebook–depression relations. Journal of Affective Disorders. 2019;248: 65–72. doi: 10.1016/j.jad.2019.01.026 30711871

[17] Kross E, Verduyn P, Sheppes G, Costello CK, Jonides J, Ybarra O. Social Media and Well-Being: Pitfalls, Progress, and Next Steps. Trends in Cognitive Sciences. 2021;25: 55–66. doi: 10.1016/j.tics.2020.10.005 33187873

[18] Beyens I, Pouwels JL, van Driel II, Keijsers L, Valkenburg PM. The effect of social media on well-being differs from adolescent to adolescent. Sci Rep. 2020;10: 10763. doi: 10.1038/s41598-020-67727-7 32612108 PMC7329840

[19] Appel H, Gerlach AL, Crusius J. The interplay between Facebook use, social comparison, envy, and depression. Current Opinion in Psychology. 2016;9: 44–49. doi: 10.1016/j.copsyc.2015.10.006

[20] Verduyn P, Ybarra O, Résibois M, Jonides J, Kross E. Do Social Network Sites Enhance or Undermine Subjective Well-Being? A Critical Review. Social Issues and Policy Review. 2017;11: 274–302. doi: 10.1111/sipr.12033

[21] Aalbers G, McNally RJ, Heeren A, de Wit S, Fried EI. Social media and depression symptoms: A network perspective. Journal of Experimental Psychology: General. 2019;148: 1454–1462. doi: 10.1037/xge0000528 30507215

[22] Valkenburg PM, Beyens I, Pouwels JL, van Driel II, Keijsers L. Social Media Browsing and Adolescent Well-Being: Challenging the “Passive Social Media Use Hypothesis.” Journal of Computer-Mediated Communication. 2021 [cited 3 Nov 2021]. doi: 10.1093/jcmc/zmab015

[23] Hardy BW, Castonguay J. The moderating role of age in the relationship between social media use and mental well-being: An analysis of the 2016 General Social Survey. Computers in Human Behavior. 2018;85: 282–290. doi: 10.1016/j.chb.2018.04.005

[24] Wirtz D, Tucker A, Briggs C, Schoemann AM. How and Why Social Media Affect Subjective Well-Being: Multi-Site Use and Social Comparison as Predictors of Change Across Time. J Happiness Stud. 2020 [cited 13 Feb 2021]. doi: 10.1007/s10902-020-00291-z

[25] Bucher T, Helmond A. The affordances of social media platforms. In: Burgess J, Marwick A, Poell T, editors. The SAGE Handbook of Social Media. London, UK: Sage Publications; 2018. pp. 233–253. doi: 10.4135/9781473984066.n14

[26] Pelletier M, Krallman A, Adams F, Hancock T. One size doesn’t fit all: a uses and gratifications analysis of social media platforms. Journal of Research in Interactive Marketing. 2020;ahead-of-print. doi: 10.1108/JRIM-10-2019-0159

[27] Cohen S, Doyle WJ, Skoner DP, Rabin BS, Gwaltney JM Jr. Social Ties and Susceptibility to the Common Cold. JAMA. 1997;277: 1940–1944. doi: 10.1001/jama.1997.03540480040036 9200634

[28] Tsai AC, Lucas M, Kawachi I. Association Between Social Integration and Suicide Among Women in the United States. JAMA Psychiatry. 2015;72: 987. doi: 10.1001/jamapsychiatry.2015.1002 26222043 PMC4598291

[29] Chin B, Cohen S. Review of the Association Between Number of Social Roles and Cardiovascular Disease: Graded or Threshold Effect? Psychosomatic Medicine. 2020;82: 471–486. doi: 10.1097/PSY.0000000000000809 32515924

[30] Ellison NB, Steinfield C, Lampe C. The Benefits of Facebook “Friends:” Social Capital and College Students’ Use of Online Social Network Sites. Journal of Computer-Mediated Communication. 2007;12: 1143–1168. doi: 10.1111/j.1083-6101.2007.00367.x

[31] Diener E, Suh ME. Subjective Well-Being and Age: An International Analysis. Annual Review of Gerontology and Geriatrics. 1997;17: 304–324. doi: 10.1891/0198-8794.17.1.304

[32] SAMHSA (Substance Abuse and Mental Health Services Administration). Key substance use and mental health indicators in the United States: Results from the 2019 National Survey on Drug Use and Health. Rockville, MD: Center for Behavioral Health Statistics and Quality, Substance Abuse and Mental Health Services Administration; 2020. Report No.: PEP20-07-01–001. Available: https://www.samhsa.gov/data/

[33] Luo Y, Waite LJ. The Impact of Childhood and Adult SES on Physical, Mental, and Cognitive Well-Being in Later Life. J Gerontol B Psychol Sci Soc Sci. 2005;60: S93–S101. doi: 10.1093/geronb/60.2.s93 15746030 PMC2505177

[34] Ko C-H, Yen J-Y, Yen C-F, Chen C-S, Chen C-C. The association between Internet addiction and psychiatric disorder: A review of the literature. European Psychiatry. 2012;27: 1–8. doi: 10.1016/j.eurpsy.2010.04.011 22153731

[35] Salmela-Aro K, Upadyaya K, Hakkarainen K, Lonka K, Alho K. The Dark Side of Internet Use: Two Longitudinal Studies of Excessive Internet Use, Depressive Symptoms, School Burnout and Engagement Among Finnish Early and Late Adolescents. J Youth Adolescence. 2017;46: 343–357. doi: 10.1007/s10964-016-0494-2 27138172

[36] Choe C, Yu S. Longitudinal Cross-Lagged Analysis Between Mobile Phone Dependence, Friendships, and Depressive Symptoms Among Korean Adolescents. Cyberpsychology, Behavior, and Social Networking. 2022 [cited 1 Jun 2022]. doi: 10.1089/cyber.2022.0015 35613406

[37] Song H, Zmyslinski-Seelig A, Kim J, Drent A, Victor A, Omori K, et al. Does Facebook make you lonely?: A meta analysis. Computers in Human Behavior. 2014;36: 446–452. doi: 10.1016/j.chb.2014.04.011

[38] van der Velden PG, Setti I, van der Meulen E, Das M. Does social networking sites use predict mental health and sleep problems when prior problems and loneliness are taken into account? A population-based prospective study. Computers in Human Behavior. 2019;93: 200–209. doi: 10.1016/j.chb.2018.11.047

[39] Allcott H, Braghieri L, Eichmeyer S, Gentzkow M. The Welfare Effects of Social Media. Rochester, NY: Social Science Research Network; 2019 Mar. Report No.: ID 3308640. doi: 10.2139/ssrn.3308640

[40] Deters F große, Mehl MR. Does Posting Facebook Status Updates Increase or Decrease Loneliness? An Online Social Networking Experiment. Social Psychological and Personality Science. 2013;4: 579–586. doi: 10.1177/1948550612469233 24224070 PMC3820167

[41] Hunt MG, Marx R, Lipson C, Young J. No More FOMO: Limiting Social Media Decreases Loneliness and Depression. Journal of Social and Clinical Psychology. 2018;37: 751–768. doi: 10.1521/jscp.2018.37.10.751

[42] Lambert J, Barnstable G, Minter E, Cooper J, McEwan D. Taking a One-Week Break from Social Media Improves Well-Being, Depression, and Anxiety: A Randomized Controlled Trial. Cyberpsychology, Behavior, and Social Networking. 2022 [cited 1 Jun 2022]. doi: 10.1089/cyber.2021.0324 35512731

[43] Przybylski AK, Nguyen TT, Law W, Weinstein N. Does Taking a Short Break from Social Media Have a Positive Effect on Well-being? Evidence from Three Preregistered Field Experiments. J technol behav sci. 2021;6: 507–514. doi: 10.1007/s41347-020-00189-w

[44] Tromholt M. The Facebook Experiment: Quitting Facebook Leads to Higher Levels of Well-Being. Cyberpsychology, Behavior, and Social Networking. 2016;19: 661–666. doi: 10.1089/cyber.2016.0259 27831756

[45] Primack BA, Shensa A, Sidani JE, Escobar-Viera CG, Fine MJ. Temporal Associations Between Social Media Use and Depression. Am J Prev Med. 2021;60: 179–188. doi: 10.1016/j.amepre.2020.09.014 33309454 PMC8261713

[46] Scott ES, Canivet C, Östergren P-O. Investigating the effect of social networking site use on mental health in an 18–34 year-old general population; a cross-sectional study using the 2016 Scania Public Health Survey. BMC Public Health. 2020;20: 1753. doi: 10.1186/s12889-020-09732-z 33225935 PMC7682097

[47] Smith TW, Davern M, Freese J, Morgan SL. General Social Surveys, 1972–2018 [machine-readable data file + codebook]. NORC at the University of Chicago; 2019.

[48] Austin PC. An Introduction to Propensity Score Methods for Reducing the Effects of Confounding in Observational Studies. Multivariate Behavioral Research. 2011;46: 399–424. doi: 10.1080/00273171.2011.568786 21818162 PMC3144483

[49] Austin PC, Stuart EA. Moving towards best practice when using inverse probability of treatment weighting (IPTW) using the propensity score to estimate causal treatment effects in observational studies. Statistics in Medicine. 2015;34: 3661–3679. doi: 10.1002/sim.6607 26238958 PMC4626409

[50] Wal WM van der, Geskus RB. ipw: An R Package for Inverse Probability Weighting. Journal of Statistical Software. 2011;43: 1–23. doi: 10.18637/jss.v043.i13

[51] Cole SR, Hernán MA, Robins JM, Anastos K, Chmiel J, Detels R, et al. Effect of Highly Active Antiretroviral Therapy on Time to Acquired Immunodeficiency Syndrome or Death using Marginal Structural Models. American Journal of Epidemiology. 2003;158: 687–694. doi: 10.1093/aje/kwg206 14507605

[52] Mahévas M, Tran V-T, Roumier M, Chabrol A, Paule R, Guillaud C, et al. Clinical efficacy of hydroxychloroquine in patients with covid-19 pneumonia who require oxygen: observational comparative study using routine care data. BMJ. 2020;369: m1844. doi: 10.1136/bmj.m1844 32409486 PMC7221472

[53] Jensen M, George MJ, Russell MR, Odgers CL. Young Adolescents’ Digital Technology Use and Mental Health Symptoms: Little Evidence of Longitudinal or Daily Linkages. Clinical Psychological Science. 2019; 2167702619859336. doi: 10.1177/2167702619859336 31929951 PMC6953732

[54] Orben A, Przybylski AK. The association between adolescent well-being and digital technology use. Nat Hum Behav. 2019;3: 173–182. doi: 10.1038/s41562-018-0506-1 30944443

[55] Przybylski AK, Murayama K, DeHaan CR, Gladwell V. Motivational, emotional, and behavioral correlates of fear of missing out. Computers in Human Behavior. 2013;29: 1841–1848. doi: 10.1016/j.chb.2013.02.014

[56] Phua J, Jin SV, Kim J (Jay). Uses and gratifications of social networking sites for bridging and bonding social capital: A comparison of Facebook, Twitter, Instagram, and Snapchat. Computers in Human Behavior. 2017;72: 115–122. doi: 10.1016/j.chb.2017.02.041

[57] DeSalvo KB, Bloser N, Reynolds K, He J, Muntner P. Mortality prediction with a single general self-rated health question. J Gen Intern Med. 2006;21: 267. doi: 10.1111/j.1525-1497.2005.00291.x 16336622 PMC1828094

[58] Schnittker J, Bacak V. The Increasing Predictive Validity of Self-Rated Health. PLOS ONE. 2014;9: e84933. doi: 10.1371/journal.pone.0084933 24465452 PMC3899056

[59] Ridgeway G, Kovalchik SA, Griffin BA, Kabeto MU. Propensity Score Analysis with Survey Weighted Data. J Causal Inference. 2015;3: 237–249. doi: 10.1515/jci-2014-0039 29430383 PMC5802372

