# Supplementary material for: Diversity of social media use: Self-selection explains associations between using many platforms and well-being
Source: PLOS Digit Health. 2023 Jul 13;2(7):e0000292. doi: 10.1371/journal.pdig.0000292 (PMC10343079; doi:10.1371/journal.pdig.0000292)
Supplement: S1 Appendix — (PDF) [file pdig.0000292.s001.pdf]

Social Media Diversity: GSS 2016 Social Media Usage module

Sophie Lohmann & Emilio Zagheni

Last updated: Juni 07, 2023

Contents

Method **1**

Supplemental Method Information 2

Results **3**

Table C **4**

Discussion **7**

Supplemental Results and Discussion 7

Note: The goal of this supplementary document is to exactly reproduce the results reported in the accompanying manuscript through the underlying R code and to generate additional analyses that do not appear in the manuscript. The document is therefore structured parallel to the manuscript.

Method

We use the 2016 wave of the General Social Survey (GSS; Smith et al., 2019) which asked N = 1372 respondents about their social media use. Participants were, on average, M = 42.94, SD = 16.00 years old, 56% were women, and 69% identified as Non-Hispanic White (14% Non-Hispanic Black, 13% Hispanic, 5% Other). Because not all questions were shown to all respondents, sample sizes differ by dependent variable (see Supplementary Table 1). However, even the sparsest variable had enough participants (n = 680) to detect effect sizes of $r \geq .11$ with 80% power.

By summing these binary variables, we derived a continuous index of social media diversity (0-14, M = 2.88, SD = 2.06).

CES-D, Cronbach's α = .76

[referring to the item on mental breakdown:] In fact, this item showed only low-to-medium correlations with all other indicators of well-being in the survey (e.g., happiness: r = -.14, health: r = -.17, depression: r = .31, bad mental health days: r = .25).

Social trust, Cronbach's α = .65

Social confidence, Cronbach's α = .78

Participants reported how many hours they spent online on a typical weekday, M = 3.13, SD = 3.43, and a typical weekend day, M = 2.93, SD = 3.42. The two variables correlated substantially with each other, r = .63, but only weakly with social media diversity, r = .18 [.13, .24] and r = .20 [.14, .27], respectively.

Supplemental Method Information

Cronbach's α for composite demographic variables: $\alpha = .63$ for the SES index, $\alpha = .85$ for the foreign-family index, $\alpha = .75$ for the religiosity index, and $\alpha = .70$ for the political conservatism index.

Age was assessed as a continuous variable in the GSS, but the oldest participants were identified only as "89 or older" in the data. We counted these values as 89 and may therefore be slightly underestimating the real mean age of the sample.

87 participants gave impossible values for the year that they had started to use the internet (i.e., before 1991), these values were subsequently set to missing.

Table 1: Table A: Sample sizes per variable

Variable	n
Bad mental health days	800
Confidence in soc. institutions	1372
Depression	680
Ever breakdown	805
Excitement about life	684
Financial satisfaction	1368
Happiness	1370
Health	686
Relationship satisfaction	728
Social trust	1370

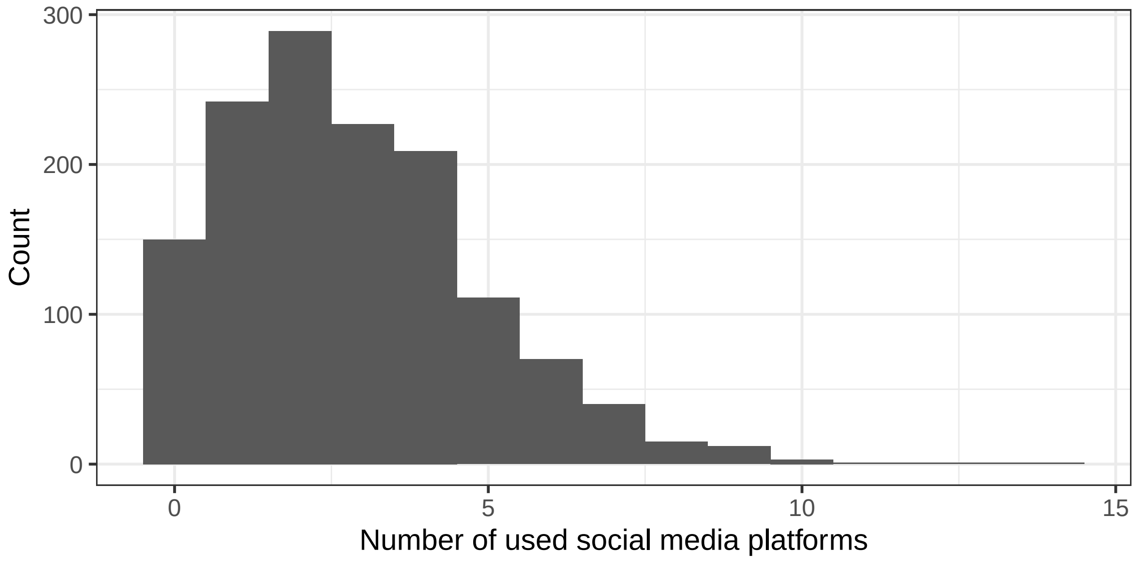

Figure 1: Figure A: Histogram of social media diversity.

Results

Table B: Linear regression results of predictor variables on number of used social media platforms

	Demographics			Demographics plus internet time use		
Term	b	p	95% CI	b	p	95% CI
(Intercept)	4.38*	< .001	[3.97, 4.80]	3.90*	< .001	[3.47, 4.32]
City size	0.00	0.053	[-0.00, 0.00]	0.00*	0.010	[0.00, 0.00]
Household size	-0.09*	0.046	[-0.18, -0.00]	-0.04	0.393	[-0.13, 0.05]
Gender: Male	-0.74*	< .001	[-0.96, -0.51]	-0.62*	< .001	[-0.84, -0.40]
Age	-0.05*	< .001	[-0.06, -0.04]	-0.03*	< .001	[-0.04, -0.02]
Nr of children	-0.04	0.397	[-0.13, 0.05]	-0.05	0.324	[-0.14, 0.04]
Race/Ethn.: Hispanic	-0.30	0.162	[-0.71, 0.12]	-0.33	0.114	[-0.75, 0.08]
Race/Ethn.: Non-Hispanic Black	0.19	0.302	[-0.17, 0.56]	0.10	0.598	[-0.27, 0.46]
Race/Ethn.: Non-Hispanic Other	0.55	0.058	[-0.02, 1.12]	0.28	0.318	[-0.27, 0.83]
SES index	0.30*	0.003	[0.10, 0.49]	0.35*	< .001	[0.16, 0.55]
Foreign family index	0.18*	0.021	[0.03, 0.33]	0.23*	0.003	[0.08, 0.38]
Religiosity index	0.12	0.089	[-0.02, 0.26]	0.06	0.366	[-0.08, 0.20]
Political conservatism index	-0.07	0.324	[-0.20, 0.07]	-0.06	0.386	[-0.19, 0.08]
Internet use start date	-0.04*	< .001	[-0.06, -0.02]	-0.03*	< .001	[-0.05, -0.02]
Region: East-South Central	-0.18	0.496	[-0.70, 0.34]	0.00	0.992	[-0.52, 0.52]
Region: Middle Atlantic	0.02	0.910	[-0.40, 0.44]	-0.01	0.979	[-0.41, 0.40]
Region: Mountain	-0.42	0.071	[-0.88, 0.04]	-0.41	0.073	[-0.86, 0.04]
Region: New England	-0.32	0.229	[-0.83, 0.20]	-0.02	0.954	[-0.54, 0.51]
Region: Pacific	-0.26	0.188	[-0.66, 0.13]	-0.10	0.613	[-0.49, 0.29]
Region: South Atlantic	-0.09	0.623	[-0.46, 0.27]	-0.03	0.866	[-0.39, 0.33]
Region: West North Central	-0.70*	0.008	[-1.21, -0.19]	-0.52*	0.047	[-1.04, -0.01]
Region: West South Central	-0.06	0.812	[-0.52, 0.41]	0.15	0.514	[-0.31, 0.61]
Internet use time on weekdays				0.03	0.217	[-0.02, 0.07]
Internet use time on weekends				0.08*	< .001	[0.03, 0.12]

Table C

Table C: Table C: Results of unadjusted linear regressions and propensity-adjusted IPTW regressions of social media diversity on z-standardized indicators of well-being. IPTW-adjusted estimates represent means and 95% percentiles of 5,000 bootstrapped samples. Maximum N = 1372 for unadjusted models, 1161 adjusted for demographic selection effects, and 1042 adjusted for demographic and internet use selection effects.

		Unadjusted		IPTW adjusted for demographics		IPTW adjusted for demographics plus internet time use	
Outcome (standardized)	Original range	b	95% CI	b	95% CI	b	95% CI
Happiness	-1 to 1	0.00	[-0.02, 0.03]	-0.02	[-0.08, 0.02]	-0.02	[-0.09, 0.03]
Health	-1 to 2	0.01	[-0.03, 0.05]	-0.03	[-0.10, 0.04]	0.03	[-0.08, 0.08]
Depression	0 to 3	0.02	[-0.02, 0.05]	0.07*	[0.00, 0.15]	0.03	[-0.03, 0.16]
Bad mental health days	0 to 30	0.03	[-0.00, 0.06]	0.08	[-0.01, 0.28]	0.01	[-0.04, 0.07]
Ever breakdown	0 to 1	0.06*	[0.03, 0.10]	0.06*	[0.00, 0.11]	0.03	[-0.04, 0.08]
Excitement about life	-1 to 1	0.04*	[0.00, 0.08]	0.01	[-0.17, 0.08]	0.01	[-0.20, 0.10]
Financial satisfaction	-1 to 1	-0.01	[-0.03, 0.02]	-0.03	[-0.08, 0.01]	-0.00	[-0.04, 0.04]
Relationship satisfaction	-1 to 1	0.03	[-0.01, 0.07]	0.03	[-0.02, 0.07]	0.03	[-0.03, 0.08]
Social trust	-1 to 1	-0.00	[-0.03, 0.02]	0.02	[-0.05, 0.08]	0.04*	[0.00, 0.10]
Confidence in soc. institutions	-1 to 1	0.03*	[0.00, 0.05]	0.05*	[0.00, 0.13]	0.03	[-0.03, 0.13]

Even without accounting for selection effects, we find few effects on adult well-being and small effect sizes. On the positive side, people who used more platforms reported finding life more exciting, $b = 0.04$ (i.e., one additional platform means an increase of 0.04 *SD*s) and more confidence in societal institutions, $b = 0.03$; on the negative side, they were more likely to have ever felt like they were going to have a nervous breakdown, $b = 0.06$.

After adjusting for the propensity to use multiple platforms based on demographic variables, the positive effect on finding life more exciting decreased to almost zero, $b = 0.01$. The remaining two effects appeared to be accounted for by time use self-selection factors such that people who generally spend more time on the internet also use more platforms: After additionally including weekday and weekend time use in the calculation of the propensity score, the negative effect of social media diversity on ever having felt like having a breakdown was halved and became non-significant, $b = 0.03$. The positive association with increased social confidence also became non-significant due to increased variance introduced by the IPTW procedure and bootstrap, although the effect size remained the same, $b = 0.03$. After accounting for self-selection involving both demographic and time use variables, the only remaining effect was a positive influence of social media diversity on increased social trust, $b = 0.04$, which had not been visible in the unadjusted results.

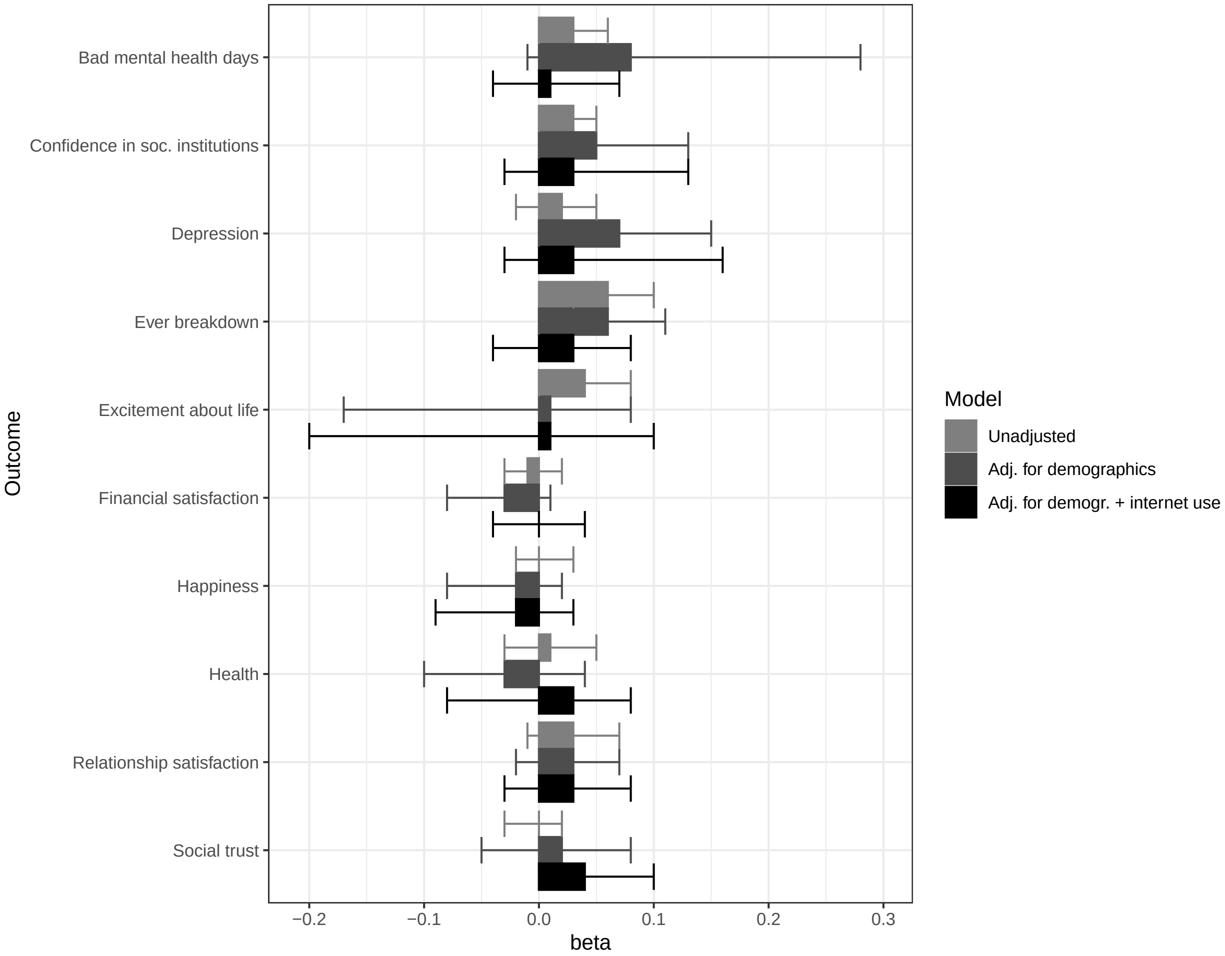

Figure 2: Figure B

Term	Happiness b	Happiness p	Health b	Health p	Depression b	Depression p	Bad mental health days b	Bad mental health days p	Ever breakdown b	Ever breakdown p	Excitement b	Excitement p	Financial satisf. b	Financial satisf. p	Relationship satisf. b	Relationship satisf. p	Trust b	Trust p	Confidence b	Confidence p
(Intercept)	4.73	0.643	-3.42	0.786	4.66	0.738	8.74	0.508	15.74	0.309	6.95	0.594	-26.46 *	0.013	5.58	0.721	42.50 *	0.000	-36.17 *	0.001
Facebook	0.00	0.979	-0.22	0.061	0.05	0.594	0.02	0.823	0.08	0.458	-0.13	0.172	-0.09	0.265	0.06	0.551	0.10	0.188	0.14 *	0.050
Twitter	-0.01	0.904	-0.02	0.865	-0.07	0.439	-0.01	0.952	-0.25	0.056	0.11	0.266	0.14	0.132	0.00	0.971	0.05	0.604	0.15 *	0.049
Tumblr	-0.49 *	0.001	-0.55 *	0.008	0.26	0.407	0.10	0.575	0.24	0.302	-0.32	0.192	-0.22	0.179	-0.31	0.195	-0.08	0.512	-0.35 *	0.006
Snapchat	0.15 *	0.041	0.18	0.148	-0.12	0.249	-0.26 *	0.009	-0.01	0.928	0.20	0.088	0.13	0.192	0.15	0.167	-0.01	0.913	0.09	0.339
Vine	0.09	0.434	-0.24	0.326	0.03	0.858	0.02	0.903	-0.05	0.791	-0.32	0.156	0.13	0.429	0.23	0.098	-0.09	0.384	0.27	0.069
Instagram	-0.13	0.127	-0.11	0.280	0.17	0.104	0.09	0.405	-0.05	0.684	-0.03	0.761	0.00	0.993	0.08	0.464	-0.04	0.546	-0.11	0.182
Pinterest	0.13	0.126	-0.02	0.849	-0.10	0.264	0.02	0.849	0.25 *	0.018	0.15	0.088	-0.01	0.898	0.02	0.839	0.06	0.365	0.01	0.935
Flickr	0.18	0.425	0.19	0.395	-0.03	0.867	0.20	0.449	0.30	0.281	0.23	0.315	-0.19	0.370	0.30	0.307	-0.08	0.702	0.23	0.343
WhatsApp	-0.00	0.970	0.25	0.065	0.16	0.208	0.04	0.767	0.17	0.354	0.13	0.275	-0.00	0.971	0.03	0.839	-0.02	0.849	-0.11	0.297
Classmates	-0.20	0.196	-0.27	0.222	0.19	0.327	0.20	0.312	0.14	0.481	0.02	0.926	-0.18	0.332	-0.25	0.152	-0.08	0.591	0.01	0.936
LinkedIn	-0.04	0.661	0.25 *	0.003	0.03	0.721	0.04	0.665	0.07	0.503	-0.07	0.442	-0.10	0.121	-0.07	0.490	0.07	0.368	-0.11	0.180
Google+	0.00	0.989	0.06	0.556	-0.03	0.753	0.00	0.978	0.06	0.493	-0.04	0.606	-0.05	0.477	0.09	0.368	0.08	0.260	0.05	0.468
City size	0.00	0.770	-0.00	0.118	-0.00	0.784	0.00	0.109	0.00	0.384	0.00	0.792	-0.00	0.933	-0.00 *	0.015	-0.00	0.537	0.00 *	0.014
Household size	0.01	0.737	-0.01	0.722	-0.08 *	0.038	0.02	0.565	0.04	0.321	0.03	0.369	0.01	0.540	0.01	0.748	0.03	0.241	0.02	0.451
Gender: Male	-0.03	0.761	-0.13	0.163	-0.11	0.232	-0.12	0.219	-0.15	0.100	0.16	0.066	0.01	0.936	0.20	0.061	0.08	0.205	0.01	0.937
Age	0.00	0.453	-0.00	0.829	-0.01 *	0.002	-0.01 *	0.027	-0.01 *	0.003	0.00	0.201	0.01 *	0.006	0.01	0.140	0.02 *	0.000	0.00	0.687
Nr of children	-0.01	0.631	-0.04	0.320	0.16 *	0.000	0.03	0.461	0.01	0.670	-0.09 *	0.015	-0.08 *	0.000	-0.16 *	0.000	-0.06 *	0.045	0.03	0.305
Race/Ethn.: Hispanic	0.15	0.126	0.40 *	0.014	-0.16	0.258	-0.07	0.679	-0.23	0.134	0.16	0.242	-0.00	0.985	-0.01	0.932	-0.04	0.758	-0.09	0.475
Race/Ethn.: Non-Hispanic Black	-0.23 *	0.016	0.15	0.323	-0.01	0.924	-0.26 *	0.018	-0.54 *	0.000	0.16	0.225	-0.22	0.068	-0.27	0.140	-0.31 *	0.003	-0.02	0.822
Race/Ethn.: Non-Hispanic Other	0.04	0.777	-0.05	0.791	0.08	0.683	-0.24	0.125	-0.24	0.203	0.19	0.233	-0.09	0.563	-0.08	0.676	-0.11	0.500	0.29	0.097
SES index	0.43 *	0.000	0.44 *	0.000	-0.38 *	0.000	-0.26 *	0.013	-0.22 *	0.006	0.35 *	0.000	0.54 *	0.000	0.00	0.969	0.30 *	0.000	0.21 *	0.000
Foreign family index	-0.03	0.497	-0.02	0.715	-0.01	0.797	-0.02	0.702	-0.07	0.369	0.04	0.420	-0.02	0.721	-0.01	0.852	-0.03	0.516	0.16 *	0.000
Religiosity index	0.07	0.066	-0.02	0.699	-0.14 *	0.015	-0.08	0.120	-0.02	0.657	0.25 *	0.000	0.05	0.231	0.10 *	0.048	0.11 *	0.004	0.07	0.105
Political conservatism index	0.04	0.361	0.09 *	0.045	-0.09 *	0.017	-0.03	0.539	-0.04	0.463	-0.05	0.283	0.01	0.691	0.06	0.196	-0.14 *	0.002	-0.11 *	0.001
Internet use start date	-0.00	0.640	0.00	0.770	-0.00	0.761	-0.00	0.512	-0.01	0.324	-0.00	0.594	0.01 *	0.013	-0.00	0.717	-0.02 *	0.000	0.02 *	0.001
Region: East-South Central	0.02	0.872	0.06	0.634	0.13	0.391	0.10	0.556	-0.01	0.956	-0.23	0.140	0.03	0.758	-0.23 *	0.049	0.03	0.804	0.07	0.298
Region: Middle Atlantic	-0.03	0.811	0.12	0.384	-0.08	0.466	0.22	0.200	-0.26	0.091	-0.12	0.385	-0.11	0.223	0.09	0.537	0.18	0.086	0.02	0.753
Region: Mountain	0.04	0.719	-0.08	0.735	-0.10	0.574	0.15	0.297	0.01	0.949	-0.16	0.440	-0.06	0.682	0.12	0.381	0.27 *	0.025	0.01	0.888
Region: New England	-0.12	0.499	0.18	0.263	-0.03	0.796	0.17	0.217	-0.46	0.077	-0.46 *	0.004	-0.03	0.787	0.18	0.187	0.07	0.430	0.03	0.701
Region: Pacific	-0.08	0.505	0.01	0.957	0.13	0.270	0.08	0.481	0.15	0.364	-0.40 *	0.025	0.01	0.939	-0.12	0.345	0.16	0.167	-0.03	0.749
Region: South Atlantic	0.01	0.946	0.13	0.149	-0.14	0.337	0.02	0.818	0.04	0.779	-0.04	0.760	0.01	0.899	0.05	0.642	0.14	0.105	0.09	0.407
Region: West North Central	-0.13	0.467	0.18	0.348	-0.08	0.423	-0.10	0.185	-0.24	0.093	-0.58 *	0.005	-0.09	0.244	0.02	0.883	-0.06	0.583	0.03	0.728
Region: West South Central	-0.06	0.726	0.13	0.395	-0.21	0.215	-0.00	0.974	-0.14	0.453	-0.18	0.207	-0.21	0.109	0.10	0.522	-0.03	0.745	0.01	0.885

Table D

Discussion

It is worth noting that none of the effects, positive or negative, unadjusted or adjusted, significant or not, were sizeable. For example, the likelihood of ever having felt like having a breakdown was increased by only 6% of a standard deviation when using one additional social media platform (the strongest effect in our unadjusted analyses) and social trust increased by only 4% of a standard deviation (the strongest effect in our fully adjusted analyses).

Supplemental Results and Discussion

The inverse-probability of treatment weights in the $Wellbeing \sim SocialMediaDiversity$ regression (output pictured in Table 2) are calculated based on propensity scores. These propensity scores in turn are derived from $SocialMediaDiversity \sim SociodemographicPredictors$ regressions. In our main analyses, propensity scores were calculated in 5000 slightly different ways (since the regression was run once for each bootstrap sample), here we present the regression results for the overall (non-bootstrapped) sample for illustration purposes.

We find that more platforms are used by women, young people, people with higher socio-economic status, and people with a more pronounced migration history in their family. People who began to use the internet in an earlier year are also likely to use more social media platforms, as are people who spend more time on the internet on weekends (but not weekdays after controlling for all other predictors). Finally, those living in larger cities, smaller households, and outside of the West North Central census division used more platforms on average.

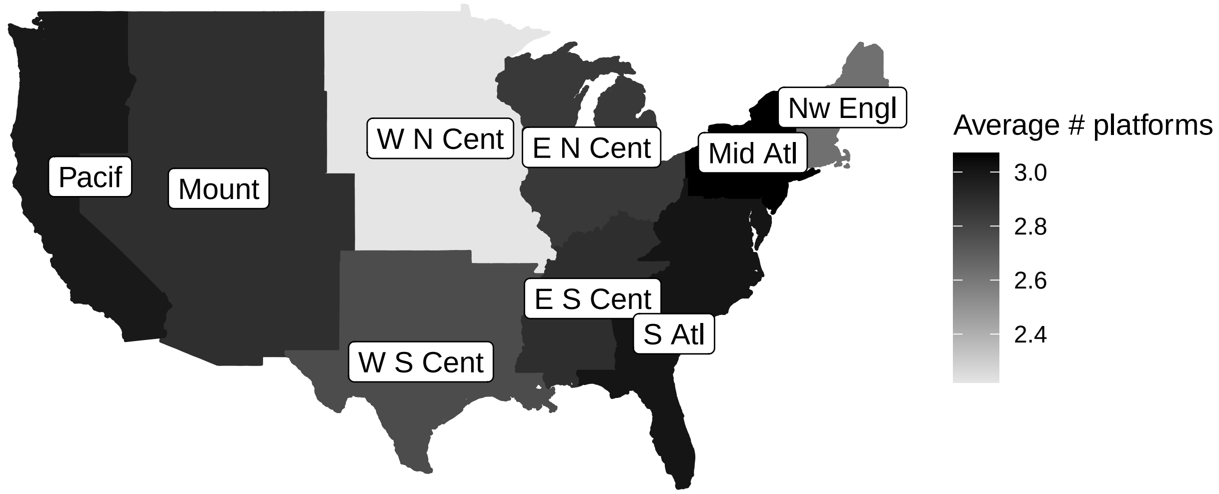

Figure 3: Figure C: Social media diversity by region
