# Supplementary material for: Diversity of social media use: Self-selection explains associations between using many platforms and well-being
Source: PLOS Digit Health. 2023 Jul 13;2(7):e0000292. doi: 10.1371/journal.pdig.0000292 (PMC10343079; doi:10.1371/journal.pdig.0000292)
Supplement: S2 Appendix — (PDF) [file pdig.0000292.s002.pdf]

Type	Variable name	Description	Question	Original coding (generally: 0 = Not applicable, 8 = Don't know, 9 = No answer)
Survey	vpsu	Variance primary sampling unit		
Survey	vstrat	Variance stratum		
Survey	wtssall	Weight variable		
Survey	ballot	Ballot used for interview		
Survey	id	Respondent id number		
Survey	year	Gss year for this respondent		
Online behavior	intuse	Use internet/apps more than occasionally	Do you use the internet or web-enabled applications, known as APPS, for more than email, at least occasionally?	1 = Yes, 2 = No
Online behavior	twitter	Use twitter	Which of the following social networking or social media sites are you a member or regular user of?	1 = Yes, 2 = No
Online behavior	facebook	Use facebook	Which of the following social networking or social media sites are you a member or regular user of?	1 = Yes, 2 = No
Online behavior	instagrm	Use instagrm	Which of the following social networking or social media sites are you a member or regular user of?	1 = Yes, 2 = No
Online behavior	linkedin	Use linkedin	Which of the following social networking or social media sites are you a member or regular user of?	1 = Yes, 2 = No
Online behavior	snapchat	Use snapchat	Which of the following social networking or social media sites are you a member or regular user of?	1 = Yes, 2 = No
Online behavior	tumblr	Use tumblr	Which of the following social networking or social media sites are you a member or regular user of?	1 = Yes, 2 = No
Online behavior	whatsapp	Use whatsapp	Which of the following social networking or social media sites are you a member or regular user of?	1 = Yes, 2 = No
Online behavior	googlesn	Use googlesn	Which of the following social networking or social media sites are you a member or regular user of?	1 = Yes, 2 = No
Online behavior	pinterst	Use pinterst	Which of the following social networking or social media sites are you a member or regular user of?	1 = Yes, 2 = No
Online behavior	flickr	Use flickr	Which of the following social networking or social media sites are you a member or regular user of?	1 = Yes, 2 = No
Online behavior	vine	Use vine	Which of the following social networking or social media sites are you a member or regular user of?	1 = Yes, 2 = No
Online behavior	clssmtes	Use clssmtes	Which of the following social networking or social media sites are you a member or regular user of?	1 = Yes, 2 = No
Online behavior	snsmoth1	Do you use any other social networks	Are there any other social networking or social media sites that you a member or regular user of?	1 = Yes, 2 = No
Online behavior	snsmot2a	First other social network	First other social network	Network
Online behavior	snsmot2b	Second other social network	Second other social network	Network
Online behavior	snsmot2c	Third other social network	Third other social network	Network
Online behavior	intstart	Year starting to use internet/apps	In what year did you start using the internet or web-enabled applications/APPS?	Year
Online behavior	intwkdym	Minutes of internet use on weekdays	How many minutes or hours do you spend actively using the Internet or web-enabled applications/APPS on a typical weekday? Minutes	Minutes
Online behavior	intwkdyh	Hours of internet use on weekdays	How many minutes or hours do you spend actively using the Internet or web-enabled applications/APPS on a typical weekday? Hours	Hours
Online behavior	intwkenm	Minutes of internet use on weekends	How many minutes or hours do you spend actively using the Internet or web-enabled applications/APPS on a typical weekeend day? Minutes	Minutes
Online behavior	intwkenh	Hours of internet use on weekends	How many minutes or hours do you spend actively using the Internet or web-enabled applications/APPS on a typical weekend day? Hours	Hours
Well-being	mntlhlth	Days of poor mental health past 30 days	Now thinking about your mental health, which includes stress, depression, and problems with emotions, for how many days during the past 30 days was your mental health not good?	Number between 0 and 30

Well-being	evbrkdwn	Ever felt going to have a nervous breakdown?	Have you ever felt that you were going to have a nervous breakdown?	1 = Yes, 2 = No
Well-being	happy	General happiness	Taken all together, how would you say things are these days--would you say that you are very happy, pretty happy, or not too happy?	1 = Very happy, 2 = Pretty happy, 3 = Not too happy
Well-being	health	Condition of health	Would you say your own health, in general, is excellent, good, fair, or poor?	1 = Excellent, 2 = Good, 3 = Fair, 4 = Poor
Well-being	life	Is life exciting or dull	In general, do you find life exciting, pretty routine, or dull?	1 = Exciting, 2 = Routine, 3 = Dull
Well-being	helpful	People helpful or looking out for selves	Would you say that most of the time people try to be helpful, or that they are mostly just looking out for themselves?	1 = Try to be helpful, 2 = Just look out for themselves, 3 = Depends (Vol.)
Well-being	fair	People fair or try to take advantage	Do you think most people would try to take advantage of you if they got a chance, or would they try to be fair?	1 = Would take advantage of you, 2 = Would try to be fair, 3 = Depends (Vol.)
Well-being	trust	Can people be trusted	Generally speaking, would you say that most people can be trusted or that you can't be too careful in dealing with people?	1 = Most people can be trusted, 2 = Can't be too careful, 3 = Other, depends (Vol.)
Well-being	cesd1	How much time felt depressed in past wk	I will now read out a list of the ways you might have felt or behaved during the past week. Using this card, please tell me how much of the time during the past week… you felt depressed?	1 = None or almost none of the time, 2 = Some of the time, 3 = Most of the time, 4 = All or almost all of the time
Well-being	cesd2	How much time sleep was restless in past wk	I will now read out a list of the ways you might have felt or behaved during the past week. Using this card, please tell me how much of the time during the past week… your sleep was restless?	1 = None or almost none of the time, 2 = Some of the time, 3 = Most of the time, 4 = All or almost all of the time
Well-being	cesd3	How much time felt happy in past wk	I will now read out a list of the ways you might have felt or behaved during the past week. Using this card, please tell me how much of the time during the past week… you were happy?	1 = None or almost none of the time, 2 = Some of the time, 3 = Most of the time, 4 = All or almost all of the time
Well-being	cesd4	How much time felt lonely in past wk	I will now read out a list of the ways you might have felt or behaved during the past week. Using this card, please tell me how much of the time during the past week… you felt lonely?	1 = None or almost none of the time, 2 = Some of the time, 3 = Most of the time, 4 = All or almost all of the time
Well-being	cesd5	How much time felt sad in past wk	I will now read out a list of the ways you might have felt or behaved during the past week. Using this card, please tell me how much of the time during the past week… you felt sad?	1 = None or almost none of the time, 2 = Some of the time, 3 = Most of the time, 4 = All or almost all of the time
Well-being	satfin	Satisfaction with financial situation	We are interested in how people are getting along financially these days. So far as you and your family are concerned, would you say that you are pretty well satisfied with your present financial situation, more or less satisfied, or not satisfied at all?	1 = Pretty well satisfied, 2 = More or less satisfied, 3 = Not satisfied at all
Well-being	hapmar	Happiness of marriage	Taking things all together, how would you describe your marriage? Would you say that your marriage is very happy, pretty happy, or not too happy?	1 = Very happy, 2 = Pretty happy, 3 = Not too happy
Well-being	hapcohab	Happiness of relt with partner	Taking things all together, would you say that your relationship with your partner is very happy, pretty happy, or not too happy?	1 = Very happy, 2 = Pretty happy, 3 = Not too happy
Well-being	confinan	Confid in banks & financial institutions	I am going to name some institutions in this country. As far as the people running these institutions are concerned, would you say you have a great deal of confidence, only some confidence, or hardly any confidence at all in them? Banks and financial institutions	1 = A great deal, 2 = Only some, 3 = Hardly any
Well-being	conbus	Confidence in major companies	I am going to name some institutions in this country. As far as the people running these institutions are concerned, would you say you have a great deal of confidence, only some confidence, or hardly any confidence at all in them? Major companies	1 = A great deal, 2 = Only some, 3 = Hardly any
Well-being	conclerg	Confidence in organized religion	I am going to name some institutions in this country. As far as the people running these institutions are concerned, would you say you have a great deal of confidence, only some confidence, or hardly any confidence at all in them? Organized religion	1 = A great deal, 2 = Only some, 3 = Hardly any
Well-being	coneduc	Confidence in education	I am going to name some institutions in this country. As far as the people running these institutions are concerned, would you say you have a great deal of confidence, only some confidence, or hardly any confidence at all in them? Education	1 = A great deal, 2 = Only some, 3 = Hardly any
Well-being	confed	Confid. in exec branch of fed govt	I am going to name some institutions in this country. As far as the people running these institutions are concerned, would you say you have a great deal of confidence, only some confidence, or hardly any confidence at all in them? Executive branch of the federal government	1 = A great deal, 2 = Only some, 3 = Hardly any

Well-being	conlabor	Confidence in organized labor	I am going to name some institutions in this country. As far as the people running these institutions are concerned, would you say you have a great deal of confidence, only some confidence, or hardly any confidence at all in them? Organized labor	1 = A great deal, 2 = Only some, 3 = Hardly any
Well-being	conpress	Confidence in press	I am going to name some institutions in this country. As far as the people running these institutions are concerned, would you say you have a great deal of confidence, only some confidence, or hardly any confidence at all in them? Press	1 = A great deal, 2 = Only some, 3 = Hardly any
Well-being	conmedic	Confidence in medicine	I am going to name some institutions in this country. As far as the people running these institutions are concerned, would you say you have a great deal of confidence, only some confidence, or hardly any confidence at all in them? Medicine	1 = A great deal, 2 = Only some, 3 = Hardly any
Well-being	contv	Confidence in television	I am going to name some institutions in this country. As far as the people running these institutions are concerned, would you say you have a great deal of confidence, only some confidence, or hardly any confidence at all in them? TV	1 = A great deal, 2 = Only some, 3 = Hardly any
Well-being	conjudge	Confid. in united states supreme court	I am going to name some institutions in this country. As far as the people running these institutions are concerned, would you say you have a great deal of confidence, only some confidence, or hardly any confidence at all in them? U.S. Supreme Court	1 = A great deal, 2 = Only some, 3 = Hardly any
Well-being	consci	Confidence in scientific community	I am going to name some institutions in this country. As far as the people running these institutions are concerned, would you say you have a great deal of confidence, only some confidence, or hardly any confidence at all in them? Scientific Community	1 = A great deal, 2 = Only some, 3 = Hardly any
Well-being	conlegis	Confidence in congress	I am going to name some institutions in this country. As far as the people running these institutions are concerned, would you say you have a great deal of confidence, only some confidence, or hardly any confidence at all in them? Congress	1 = A great deal, 2 = Only some, 3 = Hardly any
Well-being	conarmy	Confidence in military	I am going to name some institutions in this country. As far as the people running these institutions are concerned, would you say you have a great deal of confidence, only some confidence, or hardly any confidence at all in them? Military	1 = A great deal, 2 = Only some, 3 = Hardly any
Demographics	sex	Respondents sex	CODE RESPONDENT'S SEX	1 = A great deal, 2 = Only some, 3 = Hardly any
Demographics	age	Age	Respondent's age, recoded from date of birth	0 to 89 or over
Demographics	childs	Number of children	How many children have you ever had? Please count all that were born alive at any time (including any you had from a previous marriage).	0, 1, 2, 3, 4, 5, 6, 7, 8 or more
Demographics	race	Race of respondent	What race do you consider yourself? RECORD VERBATIM AND CODE. CODE WITHOUT ASKING ONLY IF THERE IS NO DOUBT IN YOUR MIND.	1 = White, 2 = Black, 3 = Other
Demographics	hispanic	Hispanic/Latino/Latina	Are you Spanish, Hispanic, or Latino/Latina?	1 = Not Hispanic, other codes = Hispanic
Demographics	born	Was r born in this country	Were you born in this country?	1 = Yes, 2 = No
Demographics	parborn	Were rs parents born in this country	Were both your parents born in this country?	0 = Both born in US, 1 = Mother yes, father no, 2 = Mother no, father yes, 3 = Mother yes, father DK, 4 = Mother no, father DK, 5 = Mother DK, father yes, 6 = Mother DK, father no, 7 = Mother DK, father DK, 8 = Neither born in US
Demographics	granborn	How many grandparents born outside u.s.	Were all of your four grandparents born in this country? IF NO: How many were born outside the United States?	0 to 4
Demographics	hompop	Number of persons in household	Household Size and Composition (see Appendix D: Recodes, for information about these variables)	open-ended number
Demographics	region	Region of interview	REGION OF INTERVIEW	1 = New England, 2 = Middle Atlantic, 3 = East North Central, 4 = West North Central, 5 = South Atlantic, 6 = East South Central, 7 = West South Central, 8 = Mountain, 9 = Pacific
Demographics	size	Size of place in 1000s	SIZE OF PLACE	population size

Demographics	wrkstat	Labor force status	Last week were you working full time, part time, going to school, keeping house, or what?	1 = Working full time, 2 = working part time, 3 = with a job, but not at work because of temporary illness, vacation, strike, 4 = unemployed, laid off, looking for work, 5 = retired, 6 = in school, 7 = keeping house
Demographics	class	Subjective class identification	If you were asked to use one of four names for your social class, which would you say you belong in: the lower class, the working class, the middle class, or the upper class?	1 = Lower class, 2 = Working class, 3 = Middle class, 4 = Upper class
Demographics	rank	Rs self ranking of social position	In our society there are groups which tend to be towards the top and those that are towards the bottom. Here we have a scale that runs from top to bottom. Where would you put yourself on this scale?	1 = Top, 2-9 are unmarked options, 10 = Bottom
Demographics	income16	Total family income	In which of these groups did your total family income, from all sources, fall last year before taxes, that is. Just tell me the letter. Hand Card A20 reads: Total income includes interest or dividends, rent, Social Security, other pension, alimony or child support, unemployment compensation, public aid (welfare), armed forces or veteran's allotment.	categories from Under $1,000, $1,000 to $2,999, $3,000 to $3,999 etc. until $170,000 or over (category breaks at 0, 1000, 3000, 4000, 5000, 6000, 7000, 8000, 10000, 12500, 15000, 17500, 20000, 22500, 25000, 30000, 35000, 40000, 50000, 60000, 75000, 90000, 110000, 130000, 150000, 170000)
Demographics	rincom16	Respondents income	In which of these groups did your earnings from (OCC), from all sources for [last year] fall? That is, before taxes or other deductions. Just tell me the letter. Hand Card A20 reads: Total income includes interest or dividends, rent, Social Security, other pension, alimony or child support, unemployment compensation, public aid (welfare), armed forces or veteran's allotment.	categories from Under $1,000, $1,000 to $2,999, $3,000 to $3,999 etc. until $170,000 or over (category breaks at 0, 1000, 3000, 4000, 5000, 6000, 7000, 8000, 10000, 12500, 15000, 17500, 20000, 22500, 25000, 30000, 35000, 40000, 50000, 60000, 75000, 90000, 110000, 130000, 150000, 170000)
Demographics	educ	Highest year of school completed	What is the highest grade in elementary school or high school that you finished and got credit for? CODE EXACT GRADE. IF FINISHED 9th-12th GRADE OR DK*: Did you ever get a high school diploma or a GED certificate? Did you complete one or more years of college for credit--not including schooling such as business college, technical or vocational school? IF YES: How many years did (you/he/she) complete? Do you have any college degrees? (IF YES: What degree or degrees?) CODE HIGHEST DEGREE EARNED.	number of years
Demographics	degree	Rs highest degree	Recode of answers to educ	0 = Less than high school, 1 = High school, 2 = Associate/Junior college, 3 = Bachelor's, 4 = Graduate
Demographics	prestg10	Rs occupational prestige score (2010)	PRESTG10 is based on the 2010 Census occupation classification. This standard prestige score is a simple mean value of ratings for each occupation category, converted to a scale of 0 (bottom) to 100 (top).	scores from 0 to 100, 0 = not applicable/no answer
Demographics	prestg105plus	Rs occupational prestige score using threshold method (2010)	PRESTG105PLUS is based on the 2010 Census occupation classification. This prestige score is calculated using an alternative method, based on the percentage of ratings that is greater than or equal to a threshold (rating five). The effect of individual raters is also removed using hierarchical linear modeling (HLM).	scores from 0 to 100, -1 = not applicable/no answer
Demographics	partyid	Political party affiliation	Generally speaking, do you usually think of yourself as a Republican, Democrat, Independent, or what?	0 = strong democrat, 1 = not very strong democrat, 2 = independent, close to democrat, 3 = independent (neither, no response), 4 = independent, close to republican, 5 = not very strong republican, 6 = strong republican
Demographics	vote12	Did r vote in 2012 election	In 2012, you remember that Obama ran for President on the Democratic ticket against Romney for the Republicans. Do you remember for sure whether or not you voted in that election?	1 = Voted, 2 = did not vote, 3 = ineligible
Demographics	pres12	Vote obama or romney	Did you vote for Obama or Romney?	1 = Obama, 2 = Romney, 3 = other candidate

Demographics	if12who	Who would r have voted for in 2012 election	Who would you have voted for, for president, if you had voted?	1 = Obama, 2 = Romney, 3 = other
Demographics	polviews	Think of self as liberal or conservative	We hear a lot of talk these days about liberals and conservatives. I'm going to show you a seven-point scale on which the political views that people might hold are arranged from extremely liberal--point 1--to extremely conservative--point 7. Where would you place yourself on this scale?	1 = extremely liberal, 2 = liberal, 3 = slightly liberal, 4 = moderate, middle of the road, 5 = slightly conservative, 6 = conservative, 7 = extremely conservative
Demographics	relig	Rs religious preference	What is your religious preference? Is it Protestant, Catholic, Jewish, some other religion, or no religion?	1 = Protestant, 2 = Catholic, 3 = Jewish, 4 = None, 5 = Other
Demographics	reliten	Strength of affiliation	Would you call yourself a strong (PREFERENCE NAMED IN RELIG) or a not very strong (PREFERENCE NAMED IN RELIG)?	1 = strong, 2 = not very strong, 3 = somewhat strong (voluntary response), 4 = no religion
Demographics	attend	How often do you attend religious services?	How often do you attend religious services? (USE CATEGORIES AS PROBES, IF NECESSARY.)	0 = Never, 1 = less than once a year, 2 = about once or twice a year, 3 = several times a year, 4 = about once a month, 5 = 2-3 times a month, 6 = nearly every week, 7 = every week, 8 = several times a week
Demographics	relactiv	How often does r take part in relig activities	How often do you take part in the activities and organizations of a church or place of worship other than attending services?	0 = Never, 1 = less than once a year, 2 = about once or twice a year, 3 = several times a year, 4 = about once a month, 5 = 2-3 times a month, 6 = nearly every week, 7 = every week, 8 = several times a week
